# Supplementary material for: Caloric Vestibular Stimulation Reduces the Directional Bias in Representational Neglect
Source: Brain Sci. 2020 May 26;10(6):323. doi: 10.3390/brainsci10060323 (PMC7348904; doi:10.3390/brainsci10060323)
Supplement: Supplementary file 1 [file brainsci-10-00323-s001.pdf]

**Supplementary material**

The following conversation with patient 3 illustrates the presence of a complete anosognosia (score 3).

Doctor: How is your left hand?

VP: *My left hand works very well. I only have a light handicap, it's my head that took a hit.*

Doctor: And your left leg?

VP: *My left leg is fine, it's all a question of practice, of controlling my effort.*

Doctor: Can you please hold out your left hand.

VP (holding out his right hand): *Here it is! It's fine.*

Doctor: What about your right hand?

VP (holding out his right hand again): *It's the same, it works fine. It has no problems*.

Doctor points to the patient's left hand.

VP: *That's my right hand, it works. Thankfully I still have that as a consolation.*

Doctor: Why a consolation?

VP: *Take a look at the state I'm in, I can't stop being depressed.*

Doctor: Why are you depressed?

VP: *Both of my parents are dead. What worries me is that I've lost my self confidence.*
